# Protective Effect of Esmolol on the Hypertensive Aortic Wall Persists After Withdrawal of Short-Term Treatment

**DOI:** 10.3390/jcm15155799

**Published:** 2026-07-24

**Authors:** David Fernández-Morales, Ana Arnalich-Montiel, María J. Delgado-Martos, Pilar Rodríguez-Rodríguez, Laia Pazó-Sayós, Raquel Martín-Oropesa, Silvia M. Arribas, Emilio Delgado-Baeza, Begoña Quintana-Villamandos

**Affiliations:** 1 Department of Anesthesiology, Lismore Base Hospital, Lismore, NSW 2480, Australia; 2 Department of Pharmacology and Toxicology, Faculty of Medicine, Universidad Complutense de Madrid, 28040 Madrid, Spain; anaarn01@ucm.es; 3 Department of Anesthesiology, Hospital General Universitario Gregorio Marañón, C/Dr. Esquerdo 46, 28007 Madrid, Spain; 4 Institute for Health Research, Hospital General Universitario Gregorio Marañón, 28007 Madrid, Spain; 5 Faculty of Experimental Sciences, Universidad Francisco de Vitoria, 28223 Madrid, Spain; 6 Department of Physiology, Faculty of Medicine, Universidad Autónoma de Madrid, 28029 Madrid, Spain

**Keywords:** aortic wall, esmolol, hypertension, spontaneously hypertensive rats, vascular remodeling

## Abstract

**Background:** Our research group has previously demonstrated the protective effect of short-term treatment with esmolol on large artery remodeling. However, whether this beneficial effect persists after treatment withdrawal remains unknown. Therefore, the aim of the present study was to investigate whether regression of thoracic aorta remodeling after a short-term esmolol treatment persists following drug withdrawal. **Methods:** Adult male spontaneously hypertensive rats (SHRs) received either esmolol (300 µg/kg/min) or vehicle (saline solution) by continuous infusion for 48 h. Following treatment, animals were evaluated either immediately (SHR-E 48 h group) or after withdrawal periods of 7 days (SHR-E 7 d group) or 1 month (SHR-E 1 m group). Hemodynamic parameters, thoracic aorta geometry, extracellular matrix composition (elastin and collagen), and the passive mechanical response of the arterial wall (β parameter) were assessed in all animals. **Results:** Forty-eight hours of esmolol treatment significantly reduced blood pressure and attenuated thoracic aortic wall thickness, external diameter, cross-sectional area, collagen content and elastic fiber density. Additionally, passive mechanical testing showed a significant reduction in the β parameter, indicating improved arterial compliance after treatment. Remarkably, these structural and mechanical benefits persisted for up to one month after withdrawal, despite the return of blood pressure to hypertensive levels. **Conclusions**: Protective effect of esmolol on aortic remodeling is persistent after treatment withdrawal in SHRs, perhaps independent of blood pressure.

## 1. Introduction

Long-term arterial hypertension leads to vascular remodeling [1]. This is associated with structural alterations of the arterial wall, including luminal narrowing, increased medial thickness, and enhanced arterial stiffness. These changes contribute to the development of cardiovascular and cerebrovascular diseases [2]. Several clinical studies have shown the beneficial effects of antihypertensive therapy (beta-blockers, calcium channel blockers, angiotensin II receptor blockers, angiotensin-converting enzyme inhibitors, and diuretics) on aortic remodeling [3], suggesting that effective blood pressure control may modify the natural progression of vascular remodeling.

Esmolol is an ultra-short-acting β1-selective adrenergic receptor blocker with a unique pharmacokinetic profile, including a half-life of approximately 9 min, rapid hydrolysis by plasma esterases, and achievement of full therapeutic effect within 5 min. These properties make it an ideal agent for perioperative hemodynamic control, minimizing the risk of hypotension and bradycardia [4]. Accordingly, esmolol is widely used in operating rooms and intensive care units (ICUs) for the management of tachyarrhythmias and arterial hypertension, including a variety of pathologies such as patients with aortic dissection [5,6].

Nebivolol and carvedilol are beta-blockers that exert protective effects on aortic remodeling by reducing oxidative stress and inhibiting vascular smooth muscle cell proliferation, respectively [7,8,9,10]. However, the vascular benefits of these agents are achieved after prolonged administration. Moreover, the protective effect of six months of nebivolol treatment persists after drug withdrawal in spontaneously hypertensive rats [11]. These findings have established the current paradigm that regression of vascular remodeling generally requires long-term antihypertensive therapy.

In contrast, our previous study demonstrated that only 48 h of esmolol administration significantly attenuated aortic thoracic remodeling in spontaneously hypertensive rats (SHR). Specifically, esmolol reduced outward hypertrophic remodeling, decreased elastic fiber volume density and improved arterial wall stiffness. These findings may have occurred as a result of a concurrent reduction in asymmetric dimethylarginine (ADMA) concentrations through attenuation of oxidative stress [12]. This phenomenon aligns with the concept of vascular memory, a paradigm suggesting that the vascular wall can retain the protective imprint of early or transient interventions against cardiovascular risk factors, including arterial hypertension, leading to sustained benefits long after the initial stimulus has ceased [13]. While this long-term legacy effect has been extensively studied in chronic settings, it remains unknown whether a highly acute pharmacological intervention can trigger a similar lasting response. Although these findings demonstrated the rapid effects of esmolol, an important physiological question remains unanswered: does such a brief pharmacological intervention induce sustained structural remodeling, or do the vessels revert to their hypertensive phenotype after esmolol withdrawal?

Answering this question is of considerable translational relevance. If a brief 48 h course of esmolol is capable of inducing sustained protection against vascular remodeling, even after drug withdrawal, this strategy could represent a clinically attractive therapeutic approach for patients with hypertension and aortic remodeling requiring short-term hemodynamic control in the operating rooms or ICUs.

Therefore, the aim of the present study was to investigate whether the regression of thoracic aorta remodeling after a short treatment with an antihypertensive drug (esmolol) persists following treatment withdrawal.

## 2. Materials and Methods

### 2.1. Ethics

The study was approved by the Ethics Review Board of Hospital General Universitario Gregorio Marañón and by the local regulatory authority (Comunidad Autónoma de Madrid), Spain (PROEX 110/17) on 31 July 2017.

All experimental procedures were conducted in compliance with European Union legislation governing the protection of animals used for experimental and other scientific purposes. The study is reported in accordance with ARRIVE guidelines.

### 2.2. Animal Experiments and Design

Rats were provided with standard laboratory chow and water ad libitum. Animals were housed under controlled environmental conditions, with a 12 h/12 h light/dark cycle, at a constant temperature of 24 °C and a relative humidity of 40%.

Animals were anesthetized with an intraperitoneal injection of diazepam and ketamine, after which a catheter was inserted into the right internal jugular vein. The catheter was subsequently protected using an iron device positioned around the neck to prevent accidental catheter removal by the animal. The external end of the catheter was then connected to an infusion system for controlled continuous intravenous administration of esmolol or saline solution.

Forty-eight male spontaneously hypertensive rats (SHRs) aged 14 months were randomly assigned to receive either intravenous esmolol (Baxter, S.L., Lessines, Belgium) (300 μg/kg/min; SHR-E, *n* = 24) or saline solution (vehicle; SHR, n = 24) for 48 h.

Animals receiving esmolol were divided into three experimental subgroups according to the time of evaluation: immediately after treatment (SHR-E 48 h, *n* = 8), 7 days after treatment withdrawal (SHR-E 7 d, *n* = 8), or 1 month after treatment withdrawal (SHR-E 1 m, *n* = 8). Likewise, vehicle-treated animals were allocated to three corresponding control groups (SHR 48 h, SHR 7 d, and SHR 1 m; *n* = 8 per group), which were evaluated at the same time points following saline infusion.

The esmolol dose was selected on the basis of our previous studies, which demonstrated its efficacy in promoting the early regression of vascular remodeling without systemic toxicity [12].

At the end of each experimental period, rats were euthanized by decapitation following previous sedation with intraperitoneal diazepam and ketamine administration. The thoracic aorta was carefully dissected and divided into consecutive segments for the assessment of vascular structure and passive mechanical properties. Investigators performing the morphometric and passive mechanical analyses were blinded to the experimental groups.

### 2.3. Blood Pressure and Heart Rate Measurements

Systolic blood pressure (SBP) and heart rate (HR) were measured before and after treatment in all animals using tail-cuff plethysmography with a photoelectric sensor (Niprem 546, Cibertec, Madrid, Spain) [12]. Briefly, rats were acclimatized to restraint cages, preheated to 37 °C for 15 min before each measurement. Three consecutive measurements were obtained from each animal for accuracy, and the mean value was used for subsequent analysis [2].

### 2.4. Histological Morphology of Thoracic Aorta

Thoracic aortic geometry was performed as previously described in similar studies [14]. One aortic segment from each animal was analyzed. For each sample and staining technique, 10 serial sections were examined, and multiple measurements from each animal were averaged before statistical analysis. Thoracic aorta segments (1 mm) were fixed in 4% sodium-buffered formaldehyde, dehydrated and embedded in paraffin. Serial 5 μm sections were stained with orcein to measure external diameter (ED) and luminal diameter (LD) of the aorta. Wall thickness (WT) was calculated as (ED–LD)/2, the wall-to-lumen ratio (W/L) as (WT/LD) × 100, and the cross-sectional area (CSA) as (π/4) × (ED2−LD2) [15]. A total of 8 thoracic aortic segments per group (SHR-E 48 h, SHR-E 7 d, SHR-E 1 m, and SHR-control groups) were analyzed using a high-resolution camera (Sony CCD IRIS, Tokyo, Japan) coupled to a Leica DMLB microscope (Wetzlar, Germany) (4× objective). Images were projected onto a computer screen for morphometric analysis.

### 2.5. Vascular Elastin and Collagen

Vascular collagen (medial and adventitia layers) and elastin (medial layer) were analyzed in eight 1 mm thoracic aortic segments per group, as previously described [12,14]. One aortic segment from each animal was analyzed. For each sample, 10 serial sections were examined, and multiple measurements from each animal were averaged before statistical analysis.

Elastic fiber content within the medial layer was compared between groups on Orcein-stained sections. The volume density of the elastic fibers was determined using a high-resolution camera (Sony CCD IRIS) attached to a Leica DMLB microscope (40× objective). Morphometric analysis was performed according to the stereological method described by Gundersen et al. [16].

Type I collagen content in the medial and adventitia layers was assessed on 5 μm paraffin sections stained with Sirius Red. Images were obtained using both light microscopy and polarized light microscopy. Collagen volume density was determined (20× objective) using the method of Gundersen et al. [16].

### 2.6. Passive Mechanical Testing

Mechanical properties of thoracic aortic rings were evaluated using an in vitro isometric recording system as previously described [12,14,17]. Descending thoracic aortic rings (3 mm in length) were mounted on two parallel intraluminal stainless steel wires and suspended in an organ bath containing an oxygenated calcium-free Krebs–Henseleit solution (0Ca2+ KHS, in mM: 115.0 NaCl, 25.0 NaHCO_3_, 4.7 KCl, 1.2 MgSO_4_, 7 H_2_O, 1.2 KH_2_PO_4_, 11.1 glucose and 10 EGTA) maintained at 37 °C. One of the wires was attached to a fixed methacrylate support, whereas the other wire was connected to an isometric force transducer. The transducer was coupled to an amplifier and a data acquisition system (Lab Chart Scope V5) (ADInstruments, Dunedin, New Zealand) in which the tension changes produced by successive stretching of the arterial segment were recorded. The distance between the wires was increased in 200 μm steps using a micrometer, and the generated force was recorded after a 3 min equilibration period at each stretch level.

For each segment, the relationship between circumferential tension and internal circumference was obtained according to the exponential equation [18] *Ti* = *ae^Bli^*, where *Ti* is the circumferential tension at each stretch level, calculated as *Ti* = *Fi*/2*g* (where *g* is the segment length (3 mm) and *Fi* is the force measured at each stretch level); *a* is a constant and *Li* is the internal circumference at each stretch level, calculated from the individual zero-tension internal diameter (obtained from the ring measurements) and the distance between the wires.

The wall stiffness parameter (β), obtained from the exponential fitting of the tension–length relationship, was used to compare the passive mechanical properties of the thoracic aorta among all experimental groups.

### 2.7. Data and Statistical Analysis

Data were expressed as mean ± standard error of the mean (SEM). Pairwise comparison between esmolol-treated and vehicle-treated animals at each point was analyzed using the independent-samples Student’s *t* test (where each experimental group represented an independent cohort of animals due to the terminal nature of the histological analysis, which required tissue collection and sacrifice at each specific stage). To control the inflation of Type I error rate, a Bonferroni correction was applied. Comparisons over time among independent cohorts within each treatment group were analyzed using one-way analysis of variance (ANOVA), followed by Bonferroni’s post hoc test when appropriate. Data normality was tested using the Shapiro–Wilk test before statistical analysis. Non-linear regression analysis based on an exponential equation model was used to estimate the mechanical stiffness parameter (β) for each individual rat. A *p*-value ≤ 0.05 was considered statistically significant. Statistical analyses were performed using IBM SPSS Statistics for Windows, version 20.0 (IBM Corp., Armonk, NY, USA).

## 3. Results

First, we investigated whether the hemodynamic effects of esmolol persisted after treatment withdrawal in SHR-E. As shown in Table 1, esmolol treatment produced a marked reduction in systolic blood pressure (SBP) and heart rate (HR). However, both parameters returned to baseline levels following treatment withdrawal.

**Figure 1:**
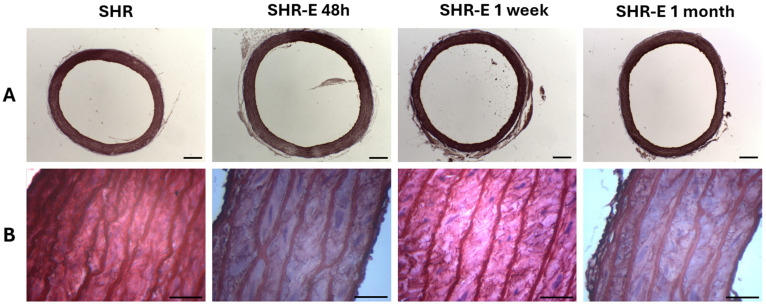
Representative photomicrographs of thoracic aortic sections stained with orcein (×40, scale bar = 150 μm) from spontaneously hypertensive rats treated with vehicle (SHR); esmolol-treated spontaneously hypertensive rats evaluated immediately after treatment (SHR-E 48 h); 7 days after drug withdrawal (SHR-E 7 d); and 1 month after drug withdrawal (SHR-E 1 m) (**A**). Representative high-magnification photomicrographs of elastic fibers in the tunica media stained with orcein (×400, scale bar = 30 μm) (**B**).

Esmolol improves thoracic aorta structure, and these beneficial effects persist after treatment withdrawal (Figure 1A). As shown in Table 2, the external diameter (ED, internal diameter + medial layer), wall thickness (WT, medial layer) and cross-sectional area (CSA, medial layer) of the thoracic aorta were significantly reduced after 48 h of esmolol treatment (SHR-E 48 h group) when compared with untreated animals (SHR 48 h group). These structural improvements were fully maintained one week after treatment discontinuation (ED, WT, CSA, SHR-E 7 d group vs. SHR 7 d group). Although these parameters gradually increased over time after treatment withdrawal, the beneficial effects of esmolol at 300 μg/kg/min were partially maintained until the end of the study (ED, WT, CSA, SHR-E 1 m group vs. SHR 1 m group). No significant differences in the luminal diameter (LD) were observed between esmolol-treated and vehicle-treated animals.

**Figure 2:**
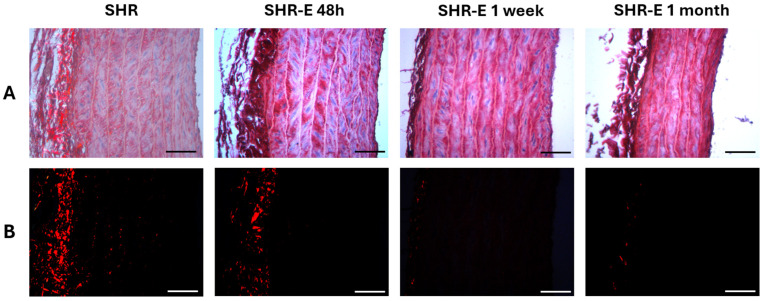
Representative photomicrographs of collagen in the medial and adventitial layers of the thoracic aorta stained with Sirius Red (×200; scale bar 60 = μm) viewed under light microscopy (**A**) and polarized light microscopy (**B**). Images are shown for spontaneously hypertensive control rats treated with vehicle (SHR), esmolol-treated spontaneously hypertensive rats evaluated immediately after treatment (SHR-E 48 h), and esmolol-treated spontaneously hypertensive rats evaluated 7 days (SHR-E 7 d) and 1 month (SHR-E 1 m) after treatment withdrawal.

Esmolol improves the elasticity of the thoracic aorta, and these beneficial effects persist after treatment withdrawal (Figure 1B). As shown in Table 3, the volume density of elastic fibers in the aorta wall (medial layer) and the volume density of collagen (medial + adventitial layers) were both significantly reduced after 48 h of esmolol treatment (SHR-E 48 h group) compared with untreated animals (SHR 48 h group) (Figure 2A,B). These changes were fully maintained one week after treatment withdrawal (SHR-E 7 d group vs. SHR 7 d group). Although both parameters gradually increased after treatment discontinuation, the beneficial effects of esmolol were partially maintained until the end of the study, with significantly lower values in the SHR-E 1 m group than in the corresponding SHR 1 m group.

Experimental circumferential wall tension–internal circumference data were fitted to an exponential model to calculate the β parameter, an index of passive arterial wall stiffness corresponding to the slope of the above curve. As shown in Table 3, the β parameter was significantly lower after 48 h of esmolol treatment (SHR-E 48 h group) compared with the corresponding control group, indicating improved arterial compliance. Notably, this beneficial effect was fully maintained for up to 1 month after treatment withdrawal (SHR-E 1 m group vs. SHR 1 m group).

## 4. Discussion

In the present study, we explored the spontaneously hypertensive rat (SHR), a well-established model of genetic hypertension that develops cardiovascular alterations comparable to those observed in human hypertension. We investigated whether regression of aortic remodeling, assessed by hemodynamic parameters, geometrical and structural features of the aortic wall, and passive aortic mechanical response, persists after withdrawal of short-term antihypertensive treatment. To our knowledge, this is the first study to demonstrate that only 48 h of treatment with the ultra-short-acting β-blocker esmolol induces sustained regression of thoracic aortic remodeling after treatment withdrawal.

Three major findings emerged from this study. First, short-term esmolol treatment significantly improved thoracic aortic structure (by reducing ED, WT and CSA). Remarkably, these structural improvements were partially maintained for up to 1 month after treatment withdrawal. Several experimental studies have previously reported similar structural changes following antihypertensive therapy in SHRs treated with losartan, captopril, enalapril, perindopril, dihydrochlorothiazide, propranolol, nifedipine, diltiazem, and verapamil [19,20,21,22,23,24,25]. However, only nifedipine and nebivolol have previously been shown to maintain vascular aortic protection after treatment discontinuation [11,23]. Seven days of nifedipine treatment attenuated abnormal aortic WT, CSA and W/L in SHR, although these morphometric changes disappeared within 24 h (CSA) and 72 h (WT) after treatment withdrawal [23]. Likewise, six months of nebivolol treatment reduced aortic WT, CSA and W/L, and this protective effect persisted for three months after treatment discontinuation [11]. In contrast, the structural benefits observed in the present study were achieved after only 48 h of esmolol administration, highlighting the rapid vascular effects of this drug.

Second, esmolol significantly reduced arterial stiffness in SHR, and this improvement persisted for one month after treatment withdrawal. Increased stiffness of large elastic arteries is a well-established predictor of cardiovascular morbidity and mortality in hypertension [26]. Numerous clinical studies have demonstrated that antihypertensive therapy (including beta-blockers, calcium channel blockers, angiotensin II receptor blockers, angiotensin-converting enzyme inhibitors, and diuretics) improves arterial stiffness in hypertensive patients, particularly after treatment periods longer than 6 months [3,27]. However, little is known about the persistence of these benefits once treatment is discontinued.

Collagen and elastin are the principal structural components of the arterial wall and play a key role in determining the elasticity and the passive mechanical properties of large elastic arteries [28,29]. Esmolol reduced elastin volume density within the medial layer, which is beneficial for maintaining aortic elasticity, and this effect was partially maintained one month after treatment withdrawal. Similar findings have been reported following four months of olmesartan treatment in SHRs, although persistence after withdrawal was not evaluated [30]. Esmolol also reduced type I collagen content in the medial and adventitial layers, and this effect also persisted after 1 month of treatment withdrawal. Because collagen is one of the major components of the adventitial layer in large arteries and directly contributes to arterial stiffening, these findings are consistent with the sustained reduction in the β parameter observed in our study [31]. Comparable reductions in collagen content have been described after six months of nebivolol treatment with effects persisting for three months after treatment discontinuation [11]. Thus, despite the marked difference in treatment duration, short-term esmolol and long-term nebivolol appear to induce comparable long-lasting improvements in vascular remodeling. Although a recent meta-analysis [27] including seventy-six studies, which evaluated the effects of antihypertensive agents in reducing arterial stiffness in patients with hypertension, suggested that this beneficial effect is largely independent of treatment duration, a more recent review of clinical studies [3] found greater improvements in aortic stiffness after antihypertensive therapy lasting longer than six months. Short-term esmolol and long-term nebivolol have a similar effect on aortic stiffness in SHR; however, further studies are required to determine first whether treatment duration influences the persistence of vascular protection after drug discontinuation, and then if these experimental findings are translatable to the clinical setting.

Finally, esmolol significantly reduced SBP in SHR, although it returned to baseline values after treatment withdrawal. These findings suggest—but do not definitively prove—that the vascular protection afforded by esmolol cannot be fully explained by blood pressure reduction and may involve blood pressure-independent mechanisms. In our previous work, esmolol reduced asymmetric dimethylarginine (ADMA) concentrations through attenuation of oxidative stress, as reflected by reduced protein carbonyl levels in the aorta [12]. Moreover, we previously demonstrated that esmolol-induced regression of coronary artery remodeling [32,33] persisted after treatment withdrawal, and was associated with sustained improvements in plasma redox status (esmolol decreased lipid peroxidation and carbonyls and improved the reduced glutathione and nitrates) [34]. These findings differ from those obtained after six months of withdrawal from nebivolol treatment, because even though SBP increased progressively, the significant differences with respect to the SHR control group persisted after three months of therapy withdrawal [11]. Nebivolol has likewise been reported to exert antioxidant and antiproliferative effects that may contribute to persistent vascular protection [35]. While the present study was not designed to directly investigate these molecular pathways, the sustained structural and mechanical improvements observed after esmolol withdrawal are directionally consistent with these previously proposed mechanisms. Nevertheless, these biochemical links remain speculative within the scope of our current data and require formal confirmation in future mechanistic studies.

Several limitations of the present study should be acknowledged. First, regarding the experimental design and model constraints, the findings of our study are restricted to the thoracic aorta of 14-month-old male SHRs (no other vascular territory or animal age was investigated). Since sex differences in cardiovascular remodeling and the anti-remodeling effects of antihypertensive therapy have been demonstrated [36,37], it would be interesting to conduct future studies to evaluate the effect of esmolol withdrawal on aortic remodeling in female hypertensive rats. Second, from a methodological standpoint, we cannot ignore the fact that the tail-cuff blood pressure method has inherent limitations compared with direct intra-arterial recordings. Consequently, although vascular protection persisted despite normalization of blood pressure, these data do not definitively establish a blood pressure-independent mechanism. In addition, the study cannot determine whether the observed histological changes truly represent permanent remodeling or reprogramming or simply a slowly temporary regression. The sample size is modest. Finally, aortic stiffness (β parameter) was evaluated under static in vitro conditions, which primarily reflect intrinsic extracellular matrix remodeling (e.g., collagen reduction). These measurements do not fully reproduce the complex hemodynamic environment present in vivo, where vascular smooth muscle tone, endothelial shear stress, pulsatile strain and neurohumoral regulation also contribute to arterial mechanics. While our results demonstrate clear structural improvements in the aortic wall, caution should be exercised when extrapolating these findings to the complex, active in vivo environment. Finally, although the SHR model is a well-established preclinical model for studying arterial hypertension, extrapolating these pathophysiological and pharmacological responses to human clinical practice must be done with caution. Further studies are needed to overcome the limitations of the present study and to elucidate the molecular pathways underlying the persistence of the beneficial effects of short-term esmolol treatment.

## 5. Conclusions

In summary, the present study provides experimental evidence that the protective effect of short-term esmolol treatment on thoracic aortic remodeling (including structure and wall stiffness) was partially maintained long-term after treatment withdrawal in SHRs. Although these findings are limited to a preclinical model, they provide a foundation for future mechanistic and translational studies exploring the concept of vascular memory induced by short-term β-blocker therapy.

## Figures and Tables

**Table 1:** Systolic blood pressure (SBP) and heart rate (HR). Data are presented as mean ± SEM (*n* = 8 samples per group). Rats treated with vehicle, SHR; rats treated with esmolol, SHR-E. * *p* < 0.05 vs. SHR; ^#^ *p* < 0.05 treatment withdrawal (7 days and 1 month) vs. treatment (48 h).

	SHR (n = 8/Group)	SHR-E (n = 8/Group)
**Heart rate (beats/min)**		
Treatment group	371 ± 17	311 ± 11 *
Withdrawal week group	383 ± 11	377 ± 10 ^#^
Withdrawal month group	379 ± 15	370 ± 14 ^#^
**Blood pressure (mmHg)**		
Treatment group	183 ± 15	140 ± 13 *
Withdrawal week group	175 ± 8	179 ± 20 ^#^
Withdrawal month group	180 ± 12	184 ± 18 ^#^

**Table 2:** Thoracic aortic geometry. Data are presented as mean ± SEM. (*n* = 8 samples per group). Spontaneously hypertensive rats treated with vehicle, SHR; spontaneously hypertensive rats treated with esmolol, SHR-E; lumen diameter, LD; external diameter, ED; wall thickness, WT; wall-to-lumen ratio, W/L; cross-sectional area, CSA. * *p* < 0.05, ** *p* < 0.01, *** *p* < 0.001 vs. SHR; ^#^ *p* < 0.05, ^##^ *p* < 0.01, ^###^ *p* < 0.001 treatment withdrawal (7 days and 1 month) vs. treatment (48 h); ^†^ *p* < 0.05, ^††^ *p* < 0.01 treatment withdrawal for 1 month vs. treatment withdrawal for 7 days.

	SHR (n = 8/Group)	SHR-E (n = 8/Group)
**LD (mm)**		
Treatment group	1.79 ± 0.05	1.74 ± 0.06
Withdrawal week group	1.83 ± 0.04	1.80 ± 0.03
Withdrawal month group	2.07 ± 0.06 ^##,††^	1.83 ± 0.11
**ED (mm)**		
Treatment group	2.18 ± 0.04	2.01 ± 0.06 *
Withdrawal week group	2.25 ± 0.05	2.07 ± 0.03 **
Withdrawal month group	2.52 ± 0.04 ^###,†^	2.19 ± 0.11 *
**WT (mm)**		
Treatment group	0.19 ± 0.008	0.13 ± 0.007 ***
Withdrawal week group	0.21 ± 0.01	0.13 ± 0.004 ***
Withdrawal month group	0.22 ± 0.01	0.17 ± 0.01 *^,#,††^
**W/L (%)**		
Treatment group	11.06 ± 0.74	7.67 ± 0.50 **
Withdrawal week group	11.52 ± 0.66	7.45 ± 0.27 ***
Withdrawal month group	11.13 ± 1.13	9.96 ± 1.22
**CSA (mm^2^)**		
Treatment group	1.21 ± 0.04	0.78 ± 0.06 ***
Withdrawal week group	1.35 ± 0.08	0.81 ± 0.03 ***
Withdrawal month group	1.64 ± 0.09 ^###^	1.13 ± 0.10 **^,#,†^

**Table 3:** Elasticity of the thoracic aorta and β parameter: density of collagen (medial and adventitial layers) and density of elastin (medial layer). Data are presented as mean ± SEM (*n* = 8 samples per group). Spontaneously hypertensive rats treated with vehicle, SHR; spontaneously hypertensive rats treated with esmolol, SHR-E. * *p* < 0.05, ** *p* < 0.01, *** *p* < 0.001 vs. SHR; ^#^ *p* < 0.05 7 days and 1 month treatment withdrawal vs. 48 h esmolol-treated group; ^†^ *p* < 0.05 1 month treatment withdrawal vs. 7 days treatment withdrawal group.

	SHR (n = 8/Group)	SHR-E (n = 8/Group)
**Density of collagen (%)**		
Treatment group	15.46 ± 1.34	4.73 ± 1.89 **
Withdrawal week group	15.42 ± 1.28	5.75 ± 0.38 ***
Withdrawal month group	19.31 ± 1.69	11.26 ± 1.68 **^,#,†^
**Density of elastin (%)**		
Treatment group	22.54 ± 1.63	8.29 ± 0.34 ***
Withdrawal week group	22.09 ± 0.72	8.84 ± 0.27 ***
Withdrawal month group	24.31 ± 1.31	11.63 ± 1.51 ***
**β parameter** **(arbitrary units)**		
Treatment group	0.85 ± 0.09	0.64 ± 0.01 *
Withdrawal week group	0.87 ± 0.08	0.65 ± 0.01 *
Withdrawal month group	0.86 ± 0.09	0.66 ± 0.007 *

## Data Availability

The datasets used and/or analyzed during the current study are available from the corresponding author on reasonable request.
